# The Role of Role Strain and Physician Authority in the Relationship Between Collaboration Orientation and Nurses’ Job Satisfaction

**DOI:** 10.1155/jonm/4015996

**Published:** 2026-09-10

**Authors:** Jingyuan Su, Lei Zhang, Tao Li

**Affiliations:** ^1^ School of Economics and Management, Zhejiang Sci-Tech University, Hangzhou, China, zstu.edu.cn; ^2^ Capital Center for Children’s Health, Capital Medical University, Capital Institute of Pediatrics, Beijing, China, shouer.com.cn

**Keywords:** collaboration orientation, job satisfaction, nurses, physician authority, role strain

## Abstract

**Aim:**

This study examined the psychological mechanism and structural boundary conditions linking nurses’ collaboration orientation with job satisfaction.

**Background:**

Nurses may value collaboration even when clinical structures limit how those values are enacted. Yet, existing research has largely focused on observable nurse–physician collaboration rather than the role‐related process through which collaborative values translate into job satisfaction. The job demands–resources model offers a useful lens, as nurses’ work attitudes are shaped by the interplay among personal resources, role‐related demands, and contextual resources. Accordingly, perceived role strain was examined as a mediating job demand and perceived physician authority as a contextual resource.

**Methods:**

A cross‐sectional survey was conducted with 566 full‐time registered nurses from tertiary and secondary hospitals in Beijing, China. All constructs were measured using scales adopted from previous studies. Data were analyzed using partial least squares structural equation modeling with 5000 bootstrap resamples to estimate direct, indirect, and moderating associations.

**Results:**

The baseline model indicated that collaboration orientation was positively associated with job satisfaction (*β* = 0.186, *p* < 0.001). Perceived role strain statistically mediated this association (indirect effect = 0.182, 95% CI [0.134, 0.232]). Perceived physician authority weakened the negative association between collaboration orientation and perceived role strain (*β* = 0.096, *p* = 0.034). The conditional indirect association between collaboration orientation and job satisfaction through perceived role strain was significantly smaller at high levels of perceived authority than at low levels of perceived physician authority (effect _high-low_ = −0.118, *p* = 0.012).

**Conclusion:**

Collaboration orientation was associated with higher job satisfaction, and this association was statistically mediated by lower perceived role strain. This indirect association was weaker when perceived physician authority was high, revealing that hierarchical authority structures may condition how collaborative values are related to nurses’ role experiences and job attitudes.

**Implications for Nursing Management:**

Nurse managers and hospital administrators may address role strain by clarifying responsibility boundaries, decision pathways, and the scope of nursing discretion. Structured nurse–physician communication and formal channels for discussing unclear role expectations may provide greater role clarity while preserving nurses’ professional voice and participation.

## 1. Introduction

Nurses routinely face multiple and sometimes competing professional expectations. In daily clinical practice, they must implement physicians’ treatment decisions, respond to patients and families, follow organizational procedures, and coordinate care with other healthcare professionals [1, 2]. These expectations do not always align. Unclear responsibility boundaries, competing priorities, and uncertainty regarding the scope of nursing discretion may make it difficult for nurses to perform their roles consistently and may affect how they evaluate their work [3].

Nurse–physician collaboration is widely considered essential to both patient care [4] and nurses’ work experiences [1, 5–8]. Because nurses and physicians contribute distinct but complementary expertise, effective patient care depends on communication, coordination, and shared responsibility between the two professions [4, 5]. However, collaborative practice is not determined solely by whether nurses value collaboration. Nurses may endorse mutual respect, shared decision‐making, and professional reciprocity even when they work in settings that provide limited opportunities to enact these values. It is therefore important to distinguish observable collaborative behavior from collaboration orientation, which reflects nurses’ beliefs about how nurse–physician work should ideally be organized [9]. Consequently, less is known about whether nurses’ underlying collaboration orientation is associated with job satisfaction and through what process this association occurs.

Valuing collaboration may provide nurses with a constructive framework for understanding interdependent clinical work, but it may not automatically lead to a positive work experience. Nurses must still reconcile expectations from physicians, patients, families, managers, and healthcare organizations [2]. When these expectations are ambiguous, inconsistent, or difficult to reconcile, nurses may experience role strain [10, 11]. Nurses with a stronger collaboration orientation may be better able to understand different professional expectations as part of an interdependent care process [12]. This may make those expectations easier to reconcile and reduce the extent to which they are experienced as fragmented or conflicting. Perceived role strain may therefore provide an important explanation for how collaboration orientation is associated with job satisfaction [13].

Unlike many occupations, nursing is embedded in a pronounced professional hierarchy in which physicians commonly hold substantial authority over clinical decisions and the allocation of responsibilities [14]. This authority structure matters not only because it determines professional influence but also because it may provide clearer decision pathways, more predictable responsibility boundaries, and greater certainty regarding accountability [15]. Perceived physician authority captures the extent to which nurses perceive physicians as controlling clinical decisions, professional discussions, and responsibility allocation. Such structural guidance may reduce nurses’ reliance on their own collaborative values when making sense of role expectations. It may also reduce the extent to which role strain is associated with dissatisfaction [16]. Examining physician authority may therefore help explain why nurses with similar collaborative orientations or similar levels of role strain report different work attitudes. However, existing studies have rarely considered these elements as parts of the same process. Research on nurse–physician collaboration has concentrated largely on observable collaborative practice, while role strain and professional authority have often been examined as separate predictors of work outcomes.

These observations indicate that nurses’ work attitudes may depend on both what they bring to the workplace and how they experience their work environment. The job demands–resources (JD‐R) model provides a useful perspective for understanding this interplay among personal resources, work demands, and contextual resources [17–20]. In this study, nursing involves substantial role‐related demands, but nurses also bring personal resources to their work and operate within organizational structures that provide contextual resources. The framework provides the overall structure for examining collaboration orientation, perceived role strain, and physician authority.

Accordingly, this study develops an integrated framework linking collaboration orientation, perceived role strain, job satisfaction, and perceived physician authority. Specifically, this study addresses two research questions: (1) whether perceived role strain mediates the relationship between collaboration orientation and nurses’ job satisfaction and (2) whether perceived physician authority conditions this psychological pathway.

This study extends research on nurse–physician collaboration beyond observable behavior by examining collaboration orientation as an internal professional resource. It identifies perceived role strain as a mechanism linking collaborative values with job satisfaction. It also places this mechanism in the professional authority structure of healthcare organizations, providing a more comprehensive understanding of how nurses’ personal orientations, role experiences, and professional contexts relate to job satisfaction.

## 2. Research Framework

### 2.1. Theoretical Foundation

The JD‐R model provides the overarching theoretical framework for this study. The JD‐R model proposes that employees’ work attitudes and well‐being are shaped by the interaction between job demands and job resources. Job demands refer to physical, psychological, social, or organizational aspects of work that require sustained effort and are therefore associated with psychological costs, such as role ambiguity, role conflict, workload, and emotional demands. Job resources refer to aspects of the work environment that facilitate goal achievement, reduce the negative consequences of job demands, and support effective functioning, including autonomy, social support, feedback, and role clarity [17, 21]. Extensions of the JD‐R model have further emphasized the role of personal resources, which refer to individuals’ beliefs and internal capacities that enable them to exert control over and effectively cope with their work environment [18]. Thus, the JD‐R model suggests that employees’ work experiences and attitudes are shaped not only by objective characteristics of the work environment but also by the personal resources they possess and their responses to workplace demands.

This framework is particularly suitable for understanding nurses’ work experiences because nursing practice involves substantial job demands arising from complex role expectations, while nurses must also draw on personal resources and operate within professional authority structures that may provide contextual resources [19]. In the context of nurse–physician relationships, collaboration orientation represents a personal resource because it reflects nurses’ internal beliefs about interprofessional work [9]. Nurses with a stronger collaboration orientation may be better equipped to maintain constructive perspectives toward nurse–physician relationships and experience more positive professional outcomes.

From a JD‐R perspective, perceived role strain represents the demanding aspect of nurses’ professional roles. It captures nurses’ perceptions of role ambiguity, role conflict, or incompatible role expectations [10, 11]. Drawing on role theory, role strain emerges when individuals must respond to inconsistent expectations from multiple role senders [22]. In nursing practice, nurses often need to invest cognitive effort to reconcile competing expectations from physicians, patients, families, and healthcare organizations. When these expectations are clear and compatible, nurses can perform their responsibilities with greater consistency and confidence; however, when expectations are difficult to reconcile, role strain is likely to occur.

However, job demands and personal resources do not operate independently of the broader work context [23–25]. Healthcare organizations are characterized by professional hierarchies, and nurses’ role experiences are embedded within unequal authority relations between occupational groups, as theorized by professional dominance theory [14, 15]. Perceived physician authority, particularly when it provides a clear and structured authority system, is conceptualized as a contextual job resource within the JD‐R model because such structural clarity can provide predictable responsibility boundaries and explicit decision‐making pathways [26]. By reducing uncertainty about who makes decisions, how responsibilities are allocated, and which procedures should be followed, physician authority provides structural clarity that can help nurses manage demanding role situations.

Combining these perspectives, the JD‐R model provides the overarching personal resource–job demand–work attitude framework, while role theory and professional dominance theory specify its operation in the nursing context. Role theory explains how incompatible expectations from multiple role senders give rise to perceived role strain, whereas professional dominance theory explains why nurses’ role experiences are embedded within physician‐led authority structures. This framework provides a theoretical foundation for explaining how nurses’ collaboration orientation is associated with job satisfaction through role experiences and why this process may vary across different authority contexts.

### 2.2. Collaboration Orientation and Job Satisfaction

The motivational process of the JD‐R model indicates that personal resources promote favorable work attitudes by supporting employees’ sense of competence, purpose, and professional meaning [27]. As a personal resource, collaboration orientation enables nurses to understand their work as part of an interdependent clinical process in which different professions contribute complementary expertise. Nurses who strongly value collaboration may consequently experience their professional roles as more meaningful and affirming [28, 29]. This positive evaluation does not require collaboration orientation to be translated into observable collaborative behavior. Rather, the value orientation itself may provide nurses with an internal source of professional meaning and a constructive basis for evaluating their work. Nurses with stronger collaboration orientation are therefore expected to report greater job satisfaction. Therefore, we propose:

 H1. Collaboration orientation is positively related to job satisfaction.

### 2.3. The Mediating Role of Perceived Role Strain

In the JD‐R framework, personal resources may help employees manage the psychological burden associated with demanding work conditions [27]. Collaboration orientation may perform this function by providing nurses with a coherent value‐based understanding of interprofessional work. From the perspective of role theory, perceived role strain arises when role expectations are ambiguous, incompatible, or difficult to reconcile [22]. However, perceived role strain may depend not only on the presence of multiple expectations but also on how nurses understand and reconcile them. Nurses with a strong collaboration orientation may be more likely to understand nurse–physician work as an interdependent professional process involving shared responsibility and professional reciprocity [29]. This role interpretation may make expectations from physicians, patients, and the organization appear more coherent and professionally meaningful, thereby being associated with lower perceived role strain. In contrast, nurses with a weaker collaboration orientation may be less likely to interpret nurse–physician work through a collaborative lens, making multiple role expectations more likely to be experienced as fragmented, imposed, or difficult to reconcile. Therefore, we propose:

 H2. Collaboration orientation is negatively related to perceived role strain.

The health‐impairment process of the JD‐R model explains why job demands require sustained psychological effort and consume employees’ mental and emotional resources. When this resource depletion persists without adequate recovery or replenishment, it may contribute to unfavorable work outcomes [30]. As a perceived role‐based job demand, perceived role strain requires nurses to cope continuously with uncertainty, confusion, and tension arising from ambiguous or conflicting role expectations. The psychological effort required to manage these conditions may gradually undermine nurses’ positive evaluations of their work, resulting in lower job satisfaction. Therefore, we propose:

 H3. Perceived role strain is negatively related to job satisfaction.

Together, these arguments suggest that collaboration orientation may influence job satisfaction through nurses’ experiences of role demands. Collaboration orientation, as a personal resource, may reduce the extent to which nurses experience their professional expectations as ambiguous or conflicting. Lower perceived role strain, in turn, may preserve more favorable evaluations of work and be associated with greater job satisfaction. Therefore, we propose:

 H4. Perceived role strain mediates the relationship between collaboration orientation and job satisfaction.

### 2.4. Perceived Physician Authority as a Moderator

The JD‐R model demonstrates that the influence of personal resources depends on the availability of contextual resources [18]. Extending the JD‐R framework, resource‐substitution reasoning proposes that when structural resources provided by the work environment are already sufficiently abundant, the marginal utility of the personal resource carried by individuals will diminish [31]. In the present study, perceived physician authority is conceptualized as a contextual job resource. Collaboration orientation as a personal resource may help nurses manage role demands by providing a collaborative framework for interpreting professional expectations. When perceived physician authority is high, the clinical environment provides stronger structural guidance regarding responsibilities and decision processes. Under such conditions, the additional contribution of collaboration orientation in reducing role strain may become less pronounced because the authority structure itself already provides clarity regarding role expectations. Conversely, when perceived physician authority is low, nurses may rely more heavily on their own collaborative orientation to interpret and manage ambiguous role expectations. Therefore, we propose:

 H5a: Perceived physician authority weakens the negative relationship between collaboration orientation and role strain such that the negative relationship is weaker when perceived physician authority is high.

The JD‐R model proposes that job resources buffer the negative effects of job demands on employee outcomes [17, 19]. In this study, perceived role strain represents a job demand, whereas perceived physician authority represents a contextual resource that may provide structural clarity and decision‐making certainty. When physician authority is perceived as high, nurses may have clearer information regarding decision responsibilities and professional boundaries. This structural resource may reduce the extent to which role strain translates into negative work evaluations because nurses have clearer reference points for understanding responsibilities and accountability. In contrast, when physician authority is low, nurses may experience greater uncertainty regarding responsibility allocation and decision processes, making role strain more psychologically burdensome. Therefore, we propose:

 H5b: Perceived physician authority weakens the negative relationship between perceived role strain and job satisfaction such that the negative relationship is weaker when perceived physician authority is high.

The moderating role of perceived physician authority in the direct relationship between collaboration orientation and job satisfaction is theoretically ambiguous. From a JD‐R perspective, competing mechanisms may exist. On one hand, a highly hierarchical authority structure may reduce the consistency between nurses’ collaborative values and their professional experiences, weakening the positive association between collaboration orientation and job satisfaction [32]. On the other hand, a clearer authority structure may provide predictable responsibilities and coordination processes, allowing collaboration‐oriented nurses to experience their work as more organized and meaningful [33]. Because these competing mechanisms show different directions, no directional hypothesis is proposed for this direct relationship. This effect is examined exploratorily.

Taken together, the JD‐R model reveals that contextual resources influence employee outcomes by shaping both the impact of personal resources on job demands and the impact of job demands on outcomes. Therefore, when perceived physician authority is high, the protective effect of collaboration orientation on role strain and the negative effect of role strain on job satisfaction are both expected to be weaker. Therefore, we propose:

 H6: Perceived physician authority weakens the indirect relationship between collaboration orientation and job satisfaction via perceived role strain such that the indirect effect is weaker when perceived physician authority is high.

**FIGURE 1:**
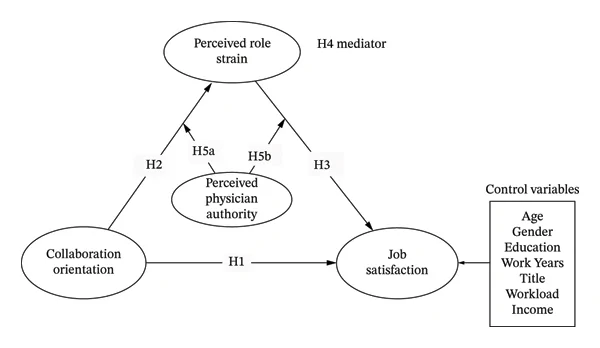
The research model.

The hypothesized model is presented in Figure 1.

## 3. Methods

### 3.1. Ethical Considerations

Ethical approval was obtained from the Institutional Review Board of Capital Center for Children’s Health, Capital Medical University (SHERLL2024018). Participation was voluntary, and all participants gave their consent before data collection. The participants were also guaranteed anonymity and confidentiality, and no personal information was collected.

### 3.2. Data Collection

This study used a cross‐sectional survey design, and data were gathered from several hospitals in Beijing, China. Beijing was chosen because it encompasses a wide range of tertiary and secondary hospitals operating within one of the most developed healthcare systems in China. As the capital city, Beijing often serves as a reference point for healthcare management and interprofessional cooperation, making it a suitable setting for research on nurse–physician relationships within a mature, well‐structured hierarchical healthcare system.

The questionnaire was designed in an electronic form by means of the Wenjuanxing platform. The research team contacted administrators of healthcare institutions to introduce the purpose of the research and obtain approval to distribute the survey. Administrators then distributed the survey link to nursing staff through several WeChat groups. To identify potential errors in online surveying, we included reverse‐coded items and ensured that participation was entirely voluntary and anonymous. The platform was also set to restrict responses to one per device/IP address to avoid repeated responses.

Participants were full‐time nurses at the time of the study and had at least 1 year of experience in their current position. To increase the response rate, periodic reminders were sent through the same WeChat groups.

Sample size adequacy was assessed using the R package WebPower with the function wp.regression() [34]. The most complex endogenous equation in the structural model was the equation predicting job satisfaction. This equation included 12 predictors, including collaboration orientation, perceived role strain, perceived physician authority, two interaction terms, and seven control variables. The analysis indicated that the required sample size was approximately 358 under an alpha level of 0.05, power of 0.80, and effect size of *f* ^2^ = 0.05.

A total of 713 responses were gathered from July to August 2024. We filtered out the invalid responses, such as those with no response variance, incomplete responses, and conflicting responses. The final valid sample of 566 responses was used for data analysis and hypothesis testing, indicating that the sample size was adequate for estimating the proposed model.

### 3.3. Measurement Instrument

The four constructs were measured using established scales and contextually adapted items, as described below. All items were assessed using a seven‐point Likert scale ranging from 1 (Strongly Disagree) to 7 (Strongly Agree). Job satisfaction was measured using Schriesheim and Tsui’s scale [35]. The scale was selected because it conceptualizes job satisfaction as employees’ evaluative judgments of work conditions and job characteristics, including supervision, compensation, professional relationships, and overall work experience. This conceptualization aligns with the present study’s focus on nurses’ cognitive evaluations of their working environment rather than affective engagement. Collaboration orientation was assessed using five representative items adapted from the Jefferson Scale of Attitudes toward Physician–Nurse Collaboration [36]. Rather than using the full scale, we selected items that specifically capture collaborative relationships between nurses and physicians, which closely align with the conceptual focus of collaboration orientation in this study. Perceived role strain was measured using five items adapted from measures of role conflict and role ambiguity developed by Rizzo et al. [10]. Perceived role strain refers to the strain and tension experienced when individuals encounter incongruent expectations, unclear responsibilities, or insufficient authority in fulfilling their organizational roles [37]. In this study, the construct was assessed within the context of nurse–physician collaboration. Perceived physician authority was assessed using three items. Two items were adapted from the physician authority dimension of the Jefferson Scale of Attitudes Toward Physician–Nurse Collaboration [36] and were reworded to reflect nurses’ perceptions of authority within their own department rather than general normative beliefs. One additional item was developed to capture the perceived dominance of physicians in patient care discussions.

Several demographic and work‐related variables were included as control variables because prior research has shown that they may influence job satisfaction [38–40]. These variables included gender, age, educational level, professional title, years of nursing experience, workload, and monthly income. Gender was coded as 1 = male and 2 = female. Age, educational level, professional title, and monthly income were measured using categorical options reflecting increasing levels. Years of nursing experience were categorized based on tenure groups. Workload was assessed by weekly working hours (≤ 45 h vs. > 45 h per week). These variables were entered into the structural model to control for their potential confounding effects on job satisfaction.

To ensure measurement validity during the translation of the instrument from English to Chinese, we utilized a back‐translation method. Three professional nurses evaluated the instrument for clarity, question wording, ease of understanding, validity, logical consistency, item sequence, and contextual relevance, recommending minor modifications to the wording and item sequence. Subsequently, a pilot study involving 40 nurses was conducted using the revised questionnaire language, which improved clarity and reduced ambiguities. We recorded the time taken by nurses to complete the survey as a criterion for evaluating response quality. Items with unsatisfactory loadings were subsequently removed. Detailed information about the survey and its items can be found in Supporting Information Table S1.

### 3.4. Statistical Analysis Methods

In the initial stage, descriptive statistics and measurement model testing were used to examine the reliability and validity of the constructs. First, descriptive statistics were calculated to summarize participants’ demographic characteristics. Second, because all constructs were measured based on nurses’ perceptions, common method variance was assessed. Harman’s single‐factor test was first conducted to examine the potential presence of common method variance. Using SPSS version 26, we examined the proportion of variance explained by the single unrotated factor [41]. Common method variance was further assessed using Kock’s full collinearity VIF approach [42]. Specifically, latent variable scores were exported from SmartPLS software Version 4.1, and each latent construct was sequentially regressed on all other latent constructs to obtain full collinearity VIF values. The recommended criterion for full collinearity VIF values is below 3.3. Third, confirmatory factor analysis using covariance‐based SEM was conducted with the lavaan package in R software to evaluate the adequacy of the measurement model by examining the *χ* ^2^/df ratio, CFI, TLI, RMSEA, and SRMR [43]. Reliability and validity were further assessed using the SmartPLS software Version 4.1. All the factor loadings were above 0.70, indicating convergent validity. Additionally, composite reliability and alpha values were also above 0.70, while the average variance extracted was above 0.50. The square root of the average variance extracted was also higher than the correlations between the constructs, supporting discriminant validity. HTMT values were below 0.85 [44, 45]. Fourth, multicollinearity was assessed using inner model VIF values. All VIF values were below the conservative threshold of 3.3 [46].

In the second stage, partial least squares structural equation modeling (PLS‐SEM) was conducted to analyze the structural model and test the proposed hypotheses [47]. Although the study was theoretically driven and confirmatory in nature, PLS‐SEM was considered appropriate because the model included direct effects, mediation, multiple moderation effects, and moderated mediation, requiring the estimation of interaction terms and conditional indirect effects. In addition, the study aimed to explain variance in perceived role strain and job satisfaction, which is consistent with the prediction‐oriented and variance‐based logic of PLS‐SEM. The bootstrapping procedure in PLS‐SEM was used to assess the statistical significance of the direct, indirect, and interaction effects without relying on strict assumptions of multivariate normality. Therefore, the structural model was analyzed using SmartPLS software Version 4.1. A bootstrapping technique with 5000 resamples was used to establish the significance of the paths. Results are reported as *R* ^2^ for the endogenous variables, standardized path coefficient (*β*), and *p*‐value. To analyze the mediating effect of perceived role strain, the bootstrapping approach was used because it does not require a normally distributed sampling distribution. Interaction terms were used to statistically analyze the moderation and moderated mediation effects. Bootstrap multigroup analysis was used to examine the moderated mediation effect [48]. The data were divided into two groups according to the mean score of perceived physician authority, creating high and low groups. Differences between these two groups indicated how perceived physician authority functioned as a moderator.

## 4. Results

### 4.1. Demographic Characteristics

Table 1 shows the demographic characteristics of the participants. The sample showed that most participants were women (95.6%) and aged 40 years or younger (90.3%). Most participants did not hold a leadership position (89.2%) and held a bachelor’s degree (65.7%). Only 27.4% had more than 10 years of nursing experience. Over half of the participants (56.5%) reported working more than 45 h per week. Most nurses were employed in governmental hospitals (72.1%). Senior nurses (37.1%) and supervisor nurses (35.5%) constituted the largest professional title groups. Monthly income was primarily concentrated in the 5001–8000 range (58.5%).

**TABLE 1:** Characteristics of participants (*n* = 566).

Demographic characteristics		Frequency	Percentage (%)
Gender	Male	25	4.4
	Female	541	95.6

Age (years)	18–25	119	21
	26–30	178	31.5
	31–40	214	37.8
	> 40	55	9.7

Education level	Junior college or below	192	33.9
	Bachelor’s degree	372	65.7
	Master’s degree or above	2	0.4

Years of nursing experience	< 6 years	202	35.7
	6–10 years	209	36.9
	> 10 years	155	27.4

Whether to hold a leadership position	No. Nurse	505	89.2
	Yes. Nursing manager	61	10.8

Professional title	Staff nurse	125	22.1
	Senior nurse	210	37.1
	Supervisor nurse	201	35.5
	Co‐chief nurse	26	4.6
	Chief nurse	4	0.7

Workload	≤ 45 h/week	246	43.5
	> 45 h/week	320	56.5

Hospital ownership	Governmental	408	72.1
	Private	158	27.9

Monthly income (CNY)	≤ 3000	20	3.5
	3001–5000	62	11.0
	5001–8000	331	58.5
	8001–10,000	134	23.7
	> 10,000	19	3.3

### 4.2. Common Method Variance

Relationships estimated using self‐report data may be subject to common method bias. Harman’s single‐factor test was performed to assess this issue. An unrotated exploratory factor analysis including all measurement items was conducted. The results revealed multiple factors with eigenvalues greater than 1, and the first factor accounted for 37.87% of the total variance. In addition, Kock’s full collinearity VIF values were all below the threshold of 3.3. Detailed results are shown in Supporting Information Table S2. Therefore, common method bias is not a serious concern in this study.

### 4.3. Measurement Model

First, the four‐factor model, including collaboration orientation (COA), perceived physician authority (PA), perceived role strain (PRS), and job satisfaction (SAT), demonstrated an acceptable overall fit to the data: *χ* ^2^(113) = 481.263, *p* < 0.001; *χ* ^2^/df = 4.259; CFI = 0.954; TLI = 0.945; RMSEA = 0.076 (90% CI: 0.069–0.083); SRMR = 0.053. These values meet commonly recommended thresholds, indicating satisfactory model fit.

Second, the values of Cronbach’s alpha, rho A, and composite (Table 2) were all greater than the threshold of 0.7, indicating good construct reliability. All the values of AVE for the constructs were greater than the threshold of 0.50 (Table 2), and each item loading on its own construct was greater than the recommended value of 0.6 (Table 3), supporting convergent validity.

**TABLE 2:** Statistics and discriminant validity.

	Alpha	rho_A	CR	AVE	1	2	3	4
1. COA	0.898	0.905	0.926	0.714	**0.845**			
2. PA	0.844	0.872	0.905	0.760	0.075^∗∗^	**0.872**		
3. PRS	0.950	0.950	0.961	0.833	−0.397^∗∗^	−0.161^∗∗^	**0.913**	
4. SAT	0.914	0.930	0.933	0.699	0.208^∗∗^	0.217^∗∗^	−0.518^∗∗^	**0.836**

*Note:* Alpha denotes Cronbach′s alpha. Bold values are the square roots of AVE; off‐diagonal values are correlation coefficients between the constructs.

Abbreviations: AVE, average variance extracted; COA, collaboration orientation; CR, composite reliability; PA, perceived physician authority; PRS, perceived role strain; SAT, job satisfaction.

^∗∗^ *p* < 0.01.

**TABLE 3:** Loadings and cross‐loadings.

	COA	PRS	PA	SAT
COA1	**0.826**	−0.319	0.081	0.260
COA2	**0.717**	−0.293	0.111	0.208
COA3	**0.888**	−0.400	0.067	0.180
COA4	**0.882**	−0.311	0.022	0.096
COA5	**0.900**	−0.335	0.029	0.113
PRS1	−0.375	**0.881**	−0.176	−0.449
PRS2	−0.399	**0.920**	−0.141	−0.474
PRS3	−0.356	**0.920**	−0.132	−0.463
PRS4	−0.363	**0.940**	−0.120	−0.485
PRS5	−0.315	**0.900**	−0.165	−0.491
PA1	0.026	−0.183	**0.896**	0.210
PA2	0.043	−0.113	**0.881**	0.191
PA3	0.149	−0.111	**0.836**	0.159
SAT1	0.295	−0.523	0.210	**0.818**
SAT2	0.192	−0.470	0.173	**0.893**
SAT3	0.135	−0.429	0.198	**0.876**
SAT4	0.136	−0.433	0.183	**0.880**
SAT5	0.041	−0.235	0.132	**0.705**
SAT6	0.173	−0.423	0.176	**0.831**

*Note:* Bold values represent loadings on the corresponding constructs, whereas nonbold values indicate cross‐loadings on other constructs.

Abbreviations: COA, collaboration orientation; PA, perceived physician authority; PRS, perceived role strain; SAT, job satisfaction.

Third, as shown in Table 2, the square root of the AVE value for each construct was greater than the construct’s correlations with other constructs, and the item loadings on their own constructs were all greater than their cross‐loadings with other constructs (Table 3). PLS results showed that all the HTMT values ranged from 0.106 to 0.538, less than the threshold of 0.85. These results supported discriminant validity.

Finally, the variance inflation factor values ranged from 1.084 to 1.383, all below the threshold of 3.3, indicating no multicollinearity issues in the current study.

### 4.4. Structural Model

Table 4 presents the results of the PLS analysis. Model 1 contained only control variables. Of the control variables, professional title (*β* = −0.174, *p* < 0.01) and workload (*β* = −0.263, *p* < 0.001) had a negative effect on job satisfaction, while physician authority (*β* = 0.164, *p* < 0.001) had a positive effect. This model explained 12.4% of the variance in job satisfaction (*R* ^2^ = 0.124).

**TABLE 4:** Summary of PLS results.

Pathways	Path coefficients (β)			
	Model 1	Model 2	Model 3	Model 4
Age ⟶ SAT	0.128	0.096	0.096	0.097
Gender ⟶ SAT	0.108	0.014	0.017	0.024
Education ⟶ SAT	0.058	0.057	0.047	0.039
Professional title ⟶ SAT	−0.174^∗∗^	−0.173^∗∗^	−0.143^∗^	−0.132^∗^
Years ⟶ SAT	−0.018	0.012	−0.023	−0.033
Workload ⟶ SAT	−0.263^∗∗∗^	−0.239^∗∗∗^	−0.171^∗∗∗^	−0.169^∗∗∗^
Monthly income ⟶ SAT	0.046	0.029	0.048	0.051
PA ⟶ SAT	0.164^∗∗∗^	0.154^∗∗∗^	0.110^∗∗^	0.096^∗^
COA ⟶ SAT	—	0.186^∗∗∗^	−0.006	0.003
PRS ⟶ SAT	—	—	−0.458^∗∗∗^	−0.457^∗∗∗^
COA ⟶ PRS	—	—	−0.397^∗∗∗^	−0.365^∗∗∗^
PA ⟶ PRS	—	—	—	−0.167^∗∗∗^
PA×COA ⟶ PRS	—	—	—	0.096^∗^
PA×PRS ⟶ SAT	—	—	—	0.079
PA×COA⟶ SAT	—	—	—	0.077
SAT *R* ^2^	0.124^∗∗∗^	0.156^∗∗∗^	0.315^∗∗∗^	0.323^∗∗∗^
SAT Δ*R* ^2^	—	0.032	0.159	0.008
*f* ^2^‐statistics	—	0.038	0.232^∗∗^	0.012

Abbreviations: COA, collaboration orientation; PA, perceived physician authority; PRS, perceived role strain; SAT, job satisfaction.

^∗^ *p* < 0.05.

^∗∗^ *p* < 0.01.

^∗∗∗^ *p* < 0.001.

Model 2 added collaboration orientation to the model. This had a positive effect on job satisfaction (*β* = 0.186, *p* < 0.001), thus supporting H1. This model explained 15.6% of the variance in job satisfaction (Δ*R* ^2^ = 0.032).

Model 3 added perceived role strain as a mediator. In this model, collaboration orientation had a negative effect on perceived role strain (*β* = −0.397, *p* < 0.001), thus supporting H2. Perceived role strain had a negative effect on job satisfaction (*β* = −0.458, *p* < 0.001), thus supporting H3. This model had a higher explanatory capability for job satisfaction (*R* ^2^ = 0.315, Δ*R* ^2^ = 0.159; *f* ^2^ = 0.232, *p* < 0.01). However, this relationship between collaboration orientation and job satisfaction lost significance when controlling for perceived role strain. In Model 3, the indirect effect was significant (effect = 0.182, 95% CI [0.134, 0.232]), thus supporting H4, as shown in Table 5. These results indicate a full mediation role of perceived role strain.

**TABLE 5:** Mediation analysis results.

Mediation effect	Model 3		Model 4	
	Effect	95% CI	Effect	95% CI
Direct effects				
COA⟶SAT	−0.006	(−0.075, 0.064)	0.003	(−0.068, 0.072)
Indirect effects				
COA⟶PRS⟶SAT	0.182^a^	(0.134, 0.232)	0.167^a^	(0.123, 0.215)
PA ×COA⟶PRS⟶ SAT	—	—	−0.044^a^	(−0.087, −0.000)
Total effects	0.176^a^	(0.101, 0.244)	0.170^a^	(0.091, 0.237)

Abbreviations: COA, collaboration orientation; PA, perceived physician authority; PRS, perceived role strain; SAT, job satisfaction.

^a^Empirical 95% CI does not overlap with zero.

Model 4 incorporated the interaction terms of physician authority. The interaction between physician authority and collaboration orientation was significant in predicting perceived role strain (*β* = 0.096, *p* = 0.034), supporting H5a. However, the interaction between physician authority and perceived role strain was not significant (*p* = 0.059) in predicting job satisfaction, nor was the interaction between physician authority and collaboration orientation (*p* = 0.147). Therefore, H5b was not supported.

As shown in Table 5, further analysis of the conditional indirect effect resulted in a significant effect (effect = −0.044, 95% CI [−0.087, −0.000]) for the moderated mediation effect in Model 4. In order to test the moderated mediation effect of physician authority, the data were divided into two groups based on the mean of physician authority, and a bootstrap multigroup analysis was performed, as shown in Table 6. The results indicated a significant difference in the two indirect effects (effect _high-low_ = −0.118, *p* = 0.012). This shows that physician authority has a mitigating effect on the indirect effect of collaboration orientation on job satisfaction mediated by perceived role strain; that is, a positive indirect effect is reduced in the presence of high physician authority. Therefore, H6 was supported.

**TABLE 6:** Bootstrap multigroup analysis for Model 3.

Mediation effect	High PA group effect	Low PA group effect	Difference (high‐low)	*p*‐value
Indirect effects	0.119^∗∗∗^	0.237^∗∗∗^	−0.118	0.012^∗^
Direct effects	0.023	−0.070	0.092	0.249
Total effects	0.142^∗^	0.167^∗∗^	−0.026	0.752

Abbreviations: COA, collaboration orientation; PA, perceived physician authority; PRS, perceived role strain; SAT, job satisfaction.

^∗^ *p* < 0.05.

^∗∗^ *p* < 0.01.

^∗∗∗^ *p* < 0.001.

## 5. Discussion

### 5.1. Principal Findings

This research investigated the psychological mechanism and structural boundary conditions linking collaboration orientation with job satisfaction. The results showed that collaboration orientation was positively related to job satisfaction indirectly through lower perceived role strain. After perceived role strain was included in the model, the direct association between collaboration orientation and job satisfaction was no longer significant, suggesting that nurses’ role experiences play a central role in explaining this relationship.

This result is consistent with previous studies showing that nurse–physician collaboration is positively associated with job satisfaction and professional commitment [3, 29]. More importantly, the present study extends this literature by shifting attention from collaboration as an organizational climate or observable behavior to collaboration orientation as a role‐related value orientation. Nurses with a stronger collaboration orientation reported lower perceived role strain, which was in turn associated with higher job satisfaction. This indicates that collaborative values may be relevant to nurses’ work attitudes because they provide a more coherent basis for understanding and reconciling interprofessional role expectations.

Nurses respond to expectations from physicians, nurse managers, patients, families, and healthcare organizations. When these expectations are ambiguous or difficult to reconcile, nurses may need to invest sustained cognitive and psychological effort, which can undermine their evaluations of work. The findings support the health‐impairment process of the JD‐R model and with role theory [21, 23]. The negative association between perceived role strain and job satisfaction is consistent with previous evidence linking role strain with dissatisfaction and emotional exhaustion [19, 33].

Perceived physician authority significantly weakened the negative association between collaboration orientation and perceived role strain. The result supports the proposed resource‐substitution explanation [31]. When physician authority is relatively low, nurses may rely more strongly on their own collaborative orientation to interpret professional expectations and manage uncertainty. When physician authority is high, the authority structure may already provide clearer decision pathways, responsibility boundaries, and accountability arrangements. Under these conditions, collaboration orientation contributes less additional value to reducing perceived role strain.

Perceived physician authority did not significantly moderate the relationship between perceived role strain and job satisfaction. Thus, the proposed buffering effect was not supported. Structural clarity may help nurses organize role expectations, but it may be less effective in reducing the negative consequences of role strain once such strain has already been experienced. At that stage, other resources, such as organizational support, professional autonomy, adequate staffing, and opportunities for role negotiation, may be more relevant [1, 49, 50]. The exploratory interaction between collaboration orientation and perceived physician authority did not significantly associate with job satisfaction. This finding demonstrates that physician authority may not directly change the value of collaboration orientation for nurses’ overall job evaluations. Physician authority therefore appeared to be more relevant to the relationship between collaboration orientation and perceived role strain than to the direct collaboration orientation–job satisfaction relationship.

The component results provide only partial support for the proposed moderation process: physician authority significantly moderated the collaboration orientation–role strain relationship, whereas its moderation of the role strain–job satisfaction relationship was not significant. Therefore, the conditional indirect association was mainly related to the weaker collaboration orientation–role strain relationship under high physician authority, rather than by simultaneous moderation of both stages.

### 5.2. Theoretical Implications

This study has several implications for existing research. First, it extends the personal resource perspective of the JD‐R model by conceptualizing collaboration orientation as a role‐related personal resource. Previous research on nurse–physician collaboration has largely focused on observable collaborative behavior or collaborative climate, both of which are partly shaped by contextual opportunities and constraints. By distinguishing collaboration orientation from actual collaborative behavior, this study highlights the theoretical relevance of nurses’ underlying professional values. A value‐based orientation may be associated with work attitudes even when it is not necessarily translated into observable collaborative behavior.

Second, this study clarifies how a personal resource may be associated with job satisfaction by integrating the JD‐R model with role theory. Collaboration orientation was associated with lower perceived role strain, which in turn was associated with higher job satisfaction. Personal resources may be related to work attitudes, not only directly, but also through how employees experience and reconcile demanding role expectations. In this way, role theory specifies the role‐related process through which the broader JD‐R resource–demand–outcome relationship operates in nursing work.

Third, the study extends the contextual resource perspective of the JD‐R model by the moderating role of physician authority. Physician authority was more relevant to the relationship between a personal resource and a perceived role demand than to the consequences of that demand. Contextual resources may operate differently across stages of the resource–demand–outcome process. This perspective also highlights that physician authority is not merely an interpersonal factor but part of the structural context in which nurses experience their roles.

### 5.3. Practical Implications

For nursing managers, improving nurses’ job satisfaction requires attention to role strain rather than focusing only on promoting collaborative values. Although collaboration training may strengthen nurses’ willingness to engage in interprofessional work collaboratively, it cannot compensate for unclear responsibilities or conflicting expectations. Managers may therefore consider clarifying responsibility boundaries between nurses and physicians, specifying professional roles, and creating opportunities to discuss ambiguous or conflicting expectations. Structured interprofessional meetings or case discussions may be useful when they provide a clearer understanding of responsibilities and coordination processes.

Managers also need to recognize that nurses’ collaborative orientation may have less influence on their experience of role strain in clinical settings characterized by stronger physician authority. This does not suggest that hierarchical authority structures should simply be strengthened; rather, it highlights the importance of ensuring that decision pathways, responsibility allocation, and communication processes are sufficiently clear. Mechanisms such as regular nurse–physician discussions, shared clarification of role boundaries, and accessible channels for addressing unclear responsibilities may help maintain structural clarity while supporting nurses’ professional participation. However, when nurses experience persistent role strain, clarifying authority structures alone may not fully address their work difficulties. In such situations, additional organizational support may be needed, such as supervisory guidance and facilitating discussions about unclear expectations, which directly address nurses’ experiences of role‐related tension.

Routine assessments of role strain, responsibility clarity, physician authority, and job satisfaction may help nursing managers identify units where organizational processes require attention. These assessments focus on work conditions rather than individual nurses. For example, units reporting high role strain may be reviewed for unclear task allocation, inconsistent instructions, or weak mechanisms for resolving interprofessional disagreements. Such assessments may help managers develop more targeted approaches to improving nurses’ work experiences.

### 5.4. Limitations and Future Research

This study has several limitations. First, the cross‐sectional design of this study does not permit causal inferences. Although the proposed model is theoretically grounded, the findings should be interpreted as associations rather than causal effects. Future longitudinal or multiple‐wave studies could help establish the temporal orderings among the variables. Second, the study relied on self‐reported data, which may raise concerns about common method variance. Although Harman’s single‐factor test and the full collinearity assessment did not show substantial common method bias, future studies could collect data from multiple sources, such as nurse supervisors, physicians, or administrative records, to further reduce this concern. Third, although the selection of hospitals in Beijing was appropriate within the Chinese healthcare context, norms and structures of professional authority may vary across cultures and healthcare systems. Future research could test the proposed model in different cultural and national contexts to further specify its boundary conditions.

## 6. Conclusion

Based on the JD‐R model, this study examined how collaboration orientation is associated with nurses’ job satisfaction through perceived role strain and whether this indirect relationship is conditioned by perceived physician authority. Collaboration orientation was associated with higher job satisfaction through lower perceived role strain. Perceived physician authority weakened the negative association between collaboration orientation and role strain, but it did not significantly buffer the association between role strain and job satisfaction. These findings underscore the importance of reducing role strain, clarifying role expectations, and creating more supportive nurse–physician interaction patterns to improve nurses’ job satisfaction.

## Author Contributions

Study design: Jingyuan Su and Lei Zhang. Data collection: Tao Li. Data analysis: Jingyuan Su and Lei Zhang. Drafting the manuscript: Jingyuan Su. Critical revisions for important intellectual content: Jingyuan Su, Lei Zhang. Obtained funding: Jingyuan Su.

## Funding

This study was supported by the National Natural Science Foundation of China (Grant No. 72301249) and by the 2025 Zhejiang Provincial Philosophy and Social Science Planning Project (Grant No. 25NDJC060YBMS).

## Conflicts of Interest

The authors declare no conflicts of interest.

## Supporting Information

Additional supporting information can be found online in the Supporting Information section.

## Supporting information


**Supporting Information** The supporting information provides additional information on the measurement instruments (Table S1) and the results of the common method bias analysis (Table S2).

## Data Availability

The data that support the findings of this study are available from the corresponding author upon reasonable request.
