# Supplementary material for: The Role of Role Strain and Physician Authority in the Relationship Between Collaboration Orientation and Nurses’ Job Satisfaction
Source: J Nurs Manag. 2026 Sep 10;2026:4015996. doi: 10.1155/jonm/4015996 (PMC13563063; doi:10.1155/jonm/4015996)
Supplement: Supplementary file 1 [file JONM-2026-4015996-s001.docx]

Supporting Information

Table S1. Measurement Scales

| Constructions | Items |
| --- | --- |
| Job satisfaction | SAT1 I am satisfied with the nature of my clinical nursing work.  SAT2 I am satisfied with my nurse manager (i.e., my immediate nursing supervisor).  SAT3 I am satisfied with my relationships with fellow nurses and physicians in this organization.  SAT4 I am satisfied with the pay I receive for my job.  SAT5 I am satisfied with the opportunities for advancement (e.g., promotion to nursing management or progression from junior to senior professional titles).  SAT6 All things considered, I am satisfied with my current job. |
| Collaboration orientation | COA1 Interprofessional relationships between physicians and nurses should be incorporated in their educational programs.  COA2 A nurse should be viewed as a collaborator and colleague of a physician rather than as an assistant.  COA3 Physicians should receive education on establishing collaborative relationships with nurses.  COA4 There are overlapping areas of responsibility between physicians and nurses.  COA5 Physicians and nurses should jointly contribute to decisions regarding patient discharge. |
| Perceived role strain | PRS1 I feel pressure arising from unclear role boundaries during joint decision-making with physicians.  PRS2 I experience role strain when required to make medical decisions beyond the nursing scope of practice (e.g., in the absence of a physician).  PRS3 I feel pressured when expressing nursing opinions due to unequal authority during discussions with physicians.  PRS4 I experience role tension when asked to provide input on critical decisions without possessing corresponding authority.  PRS5 I feel pressure due to role boundary constraints when disagreements with physicians over treatment plans occur. |
| Perceived physician authority | PA1 In my department, nurses are generally expected to carry out physicians’ orders.  PA2 In my department, physicians typically hold dominant authority in clinical decision-making.  PA3 In discussions regarding patient care, physicians' opinions carry the final authority. |

Table S2 Kock’s full collinearity VIF values

| Dependent Variables | Independent Variables | | | |
| --- | --- | --- | --- | --- |
|  | COA | PRS | PA | SAT |
| COA | - | 1.37 | 1.052 | 1.401 |
| PRS | 1.046 | - | 1.05 | 1.091 |
| PA | 1.187 | 1.551 | - | 1.366 |
| SAT | 1.187 | 1.211 | 1.026 | - |

Note: COA, collaboration orientation; PA, perceived physician authority; PRS, perceived role strain; SAT, job satisfaction.
